# Ricin Trafficking in Plant and Mammalian Cells

**DOI:** 10.3390/toxins3070787

**Published:** 2011-06-30

**Authors:** J. Michael Lord, Robert A. Spooner

**Affiliations:** School of Life Sciences, University of Warwick, Coventry CV4 7AL, UK; Email: r.a.spooner@warwick.ac.uk

**Keywords:** ricin biosynthesis, anterograde transport, retrograde transport, endoplasmic reticulum, retrotranslocation

## Abstract

Ricin is a heterodimeric plant protein that is potently toxic to mammalian and many other eukaryotic cells. It is synthesized and stored in the endosperm cells of maturing *Ricinus communis* seeds (castor beans). The ricin family has two major members, both, lectins, collectively known as *Ricinus communis* agglutinin ll (ricin) and *Ricinus communis* agglutinin l (RCA). These proteins are stored in vacuoles within the endosperm cells of mature *Ricinus* seeds and they are rapidly broken down by hydrolysis during the early stages of post-germinative growth. Both ricin and RCA traffic within the plant cell from their site of synthesis to the storage vacuoles, and when they intoxicate mammalian cells they traffic from outside the cell to their site of action. In this review we will consider both of these trafficking routes.

## 1. Introduction

Ricin and RCA are galactose-specific lectins, each possessing at least two sugar binding sites. Ricin was, in fact, the first lectin to be described well over a century ago [1]. Stillmark, a PhD student at the University of Dorpat in Estonia, was looking for an explanation for the well known toxicity of *Ricinus* seed extracts. He studied the effects of mixing seed extract with blood and observed that the red blood cells began to agglutinate. He established that the active seed component was a protein which he termed ricin. We now know that the agglutination he observed is largely due to RCA, which is a strong haemagglutinin but a weak cytotoxin, whereas ricin is a weak haemagglutinin but is potently cytotoxic [2,3]. This results from ricin having a single B chain, enabling it to bind to and enter target cells, whereas RCA has two B chains allowing it to bind to, and thus agglutinate, two target cells.

Ricin is a heterodimer in which a catalytically-active A chain (ricin toxin A or RTA) is joined by a single disulfide bond to a B chain (RTB) that is a galactose- and *N*-acetylgalactosamine-specific lectin. The A chain is an enzyme that removes a specific adenine residue from the 28S ribosomal RNA (28SrRNA) of the large subunit of eukaryotic ribosomes [4]. The adenine residue removed by RTA, adenine 4324 in the case of rat liver 28SrRNA, is located in a region of the rRNA that contains one of the most conserved of rRNA sequences. This region functions in the ribosomal elongation cycle, and the adenine removed as a result of RTA-catalysed depurination is the binding site for elongation factors 1 and 2 [5]. Since RTA-modified ribosomes are unable to bind these translation factors they are no longer capable of continuing protein synthesis. This ultimately leads to cell death and accounts for the extreme cytotoxicity of ricin [6,7,8]. RCA is a tetramer of two ricin-like heterodimers, each of which consists of an A and a B chain. The primary sequence of the A chains of ricin and RCA are identical in all but 18 positions (from a total of 267 amino acids) and are thus 93% homologous. The corresponding B chains differ at 41 residues (from a total of 262 residues) and are 84% homologous [9,10]. Two of the 18 residue differences between RTA and RCA A chain involve the substitution of cysteine residues, one of which (Cys156) forms a disulfide bond with an adjacent molecule to generate the mature ~128 kDa tetrameric RCA with the subunit arrangement B-A-A-B [11,12].

## 2. Ricin Trafficking In Plant Cells

### 2.1. Ricin Biosynthesis

Ricin and RCA are synthesized in the seeds at the developmental stage when seed storage proteins are being synthesized and, like these storage proteins, the lectins are located in the storage vacuoles of the mature seed [13,14]. Lectin and storage protein synthesis is therefore both developmentally regulated and tissue specific, with synthesis occurring exclusively in the endosperm cells of maturing seeds [15]. Here they accumulate in protein storage vacuoles until, at seed dessication, the lectins account for approximately 5% of the total particulate protein [13,14]. The storage proteins are hydrolysed during the first few days after germination to provide a source of amino acids for the synthesis of proteins encoded by genes that are expressed at this stage of seedling development. After a few days of post-germinative growth, the stored protein reserves, including ricin and RCA, have disappeared entirely as the developing plant is increasingly able to synthesize the amino acids it needs via photosynthesis. The ricin gene family (which includes both ricin and RCA) has been reported to contain 6–8 members, as detected by Southern blot analysis using ricin cDNA probes [16,17]. However, the recently reported draft genome sequence of *Ricinus communis* revealed 28 putative genes in the family, including potential pseudogenes or gene fragments, but only one copy of the gene set responsible for castor oil biosynthesis, suggesting that there is a selective pressure for the seeds to produce a highly toxic protein [18]. The biological function of ricin therefore appears to be as a seed storage protein, presumably with the added advantage that its potent toxicity deters herbivores from eating the seeds.

### 2.2. Synthesis and Endoplasmic Reticulum Translocation

Heterodimeric ricin is initially synthesized as a single chain polypeptide precursor (preproricin) containing both the RTA and RTB sequences [9]. Preproricin contains 576 amino acid residues, the first 35 of which encode a 26 residue *N*-terminal signal sequence and a 9 residue propeptide [19], followed by the mature RTA sequence (267 residues) joined by a 12 residue linker peptide to the mature RTB sequence (262 residues). The 12 residue linker peptide contains a targeting signal that directs the ricin precursor to the vacuole [20]. During synthesis in the plant cell, the *N*-terminal signal sequence directs the transport of the nascent polypeptide across the endoplasmic reticulum (ER) membrane and into the ER lumen, leaving the 9 residue propeptide exposed at the *N*-terminus. 

Translocation across the ER membrane is accompanied by three major modifications. First, the *N*-terminal signal sequence is co-translationally cleaved by ER luminal signal peptidase. Second, proricin is *N*-glycosylated as it enters the ER lumen. Proricin has four *N*-glycosylation sites, two within the RTA sequence and a further two in RTB [21]. Third, protein disulfide isomerase catalyses the formation of five disulfide bonds in the folding protein. In mature ricin, four of these disulfide bonds are within RTB, while the fifth one is the disulfide linking RTA to RTB. The individual heterodimers that constitute RCA are synthesised as preproRCA in an identical manner to preproricin [10] but, as noted above, RCA contains an additional disulfide bond that links two heterodimers to form a tetramer [11].

The 9 residue propeptide at the *N*-terminus of RTA after cleavage of the signal peptide acts as a spacer that influences the efficiencies of both co-translational transport across the ER membrane and core glycosylation [22]. Newly synthesized proricin in the ER lumen lacks the 26 residue signal peptide, which was cleaved co-translationally, but still includes the *N*-terminal propeptide and the 12 residue linker peptide. This is probably important for the producing plant whose own ribosomes are susceptible to the action of RTA [23], because the RTA moiety of proricin, in contrast to free RTA, is catalytically inactive [24]. Association with the B chain inhibits the catalytic activity of the A chain. This inactivity results from a steric hindrance of the RTA active site by RTB in the proricin precursor [24]. Indeed, this inhibition of the RTA active site by RTA:RTB coupling persists in the mature heterodimer [25]. Since ricin is produced, transported and stored as a catalytically-inactive form, should any precursor be inefficiently or incompletely imported the hindered active site would prevent ribosome inactivation.

### 2.3. Anterograde Trafficking in the Plant Cell

**Figure 1:**
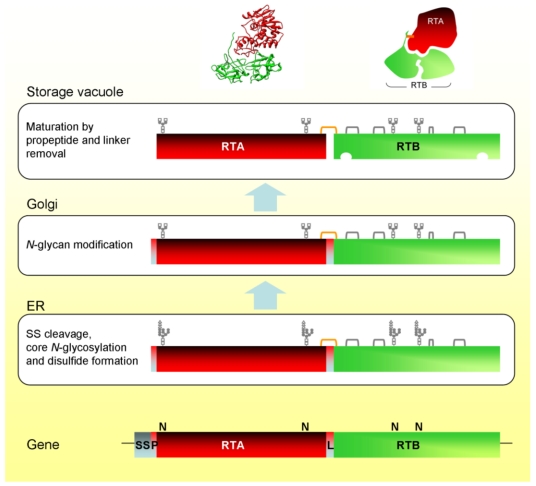
Biosynthesis and intracellular trafficking of ricin and its precursors in *Ricinus* endosperm cells. SS, signal sequence; P, propeptide; L, linker peptide; orange bracket, interchain disulfide; gray brackets, intrachain disulfides. Also shown is the ricin X-ray structure and a cartoon showing the arrangement of the chains and the position of the interchain disulfide bond.

Glycosylated proricin is transported in vesicular carriers from the ER, via the Golgi complex, to the vacuole. The attached glycans are modified during intracellular transport [21,26] and, upon reaching the vacuole, proricin is processed by an endopeptidase that removes the 12 residue linker sequence between RTA and RTB, and the 9 residue *N*-terminal propeptide [21]. The vacuolar processing enzyme (VPE) responsible for cleaving proricin does so on the *C*-terminal side of asparagine residues. Both of the peptides removed from proricin terminate in asparagine. This type of proteolysis is typical in the maturation of seed storage proteins [27,28,29]. VPE has also been identified as a caspase involved in vacuole-mediated cell death [30]. RTA and RTB are still covalently joined by a disulfide bond because cleavage of the linker occurs within a disulfide loop connecting the A and B chain sequences [31]. The biosynthesis and intracellular transport of ricin in *Ricinus* cells is illustrated schematically in Figure 1.

The ricin biosynthetic events in *Ricinus* endosperm have been recapitulated in tobacco protoplasts using transient expression and metabolic labelling [32]. This provided the first indication that the 12 residue linker peptide contained the vacuolar sorting signal. The linker peptide contains the amino acid sequence LLIRP, which is reminiscent of the sequence specific vacuolar sorting signal NPIRL found in proteins such as sweet potato sporamin [33] and barley aleurain [34]. Replacing the isoleucine of this sequence with glycine resulted in the complete secretion of proricin [20].

## 3. Ricin Trafficking in Mammalian Cells

In order to reach its ribosomal target in mammalian cells, free RTA must ultimately be delivered into the cytosol of such cells. RTA, in common with the catalytic moieties of several other protein toxins, is believed to achieve the ultimate step of entry into the cytosol from the lumen of the ER by subverting a quality control process in the ER known as ER-associated protein degradation (ERAD) [35]. Before discussing how this is thought to be achieved, here is a brief description of the current understanding of the process of ERAD.

In eukaryotic cells, the ER is the site of entry for proteins destined for the secretory pathway or for insertion into the membranes of organelles involved in this pathway. The nascent proteins enter the ER lumen via the Sec61 translocon present in the ER membrane [36]. In the ER lumen these newly synthesised proteins fold and mature, processes that depend on ER chaperones and may include ER-located enzymes that catalyse core *N*-glycosylation or disulfide bond formation. Oligomeric proteins are also assembled from their monomeric constituents. The fidelity of these processes is monitored by an ER quality control (ERQC) surveillance. Newly synthesised proteins that fold or assemble correctly are permitted by ERQC to remain in the ER (if this compartment is their ultimate destination) or to exit the ER in vesicular carriers if destined for post-ER compartments or for secretion from the cell. Proteins that fail to fold or assemble correctly are recognized as such and disposed of, since their accumulation in the cell would be detrimental. Such proteins are disposed of by the aspect of ERQC known as ERAD [37].

ERAD involves multiple disposal processes which remove the aberrant proteins from the ER by directing them to the cytosolic proteasomes for proteolytic degradation [38]. Removal from the ER (known as retrotranslocation or dislocation) requires ER membrane ubiquitin ligase complexes that polyubiquitylate the target ERAD substrates as they are extruded through the ‘dislocon’ to enter the cytosol [39,40]. Polyubiquitylation targets the ERAD substrates to the AAA-ATPase p97 (Cdc48 in yeast) complex that is the extraction motor [41,42,43,44] that delivers them to the proteasome [45,46]. If the dislocated proteins are glycosylated, they are de-glycosylated, and all ERAD substrates are de-ubiquitylated before being destroyed by proteasomal degradation [38].

Complete destruction of ERAD substrates by the proteasome does not appear to be an unavoidable event, and a few ER-located proteins appear to subvert ERAD by uncoupling, at least in part, from the final destructive step. For example, the human hepatitis E virus ORF2 protein is initially present in the ER but it subsequently appears in the cytosol, where it refolds rather than being degraded [47]. Firefly and *Renilla* luciferases internalized by dendritic cells are thought to traffic in vesicular structures to reach the ER where they unfold to subvert ERAD and subsequently refold after reaching the cytosol [48]. In addition, calreticulin, normally a resident protein of the ER, has been identified in the mammalian cell cytosol. This cytosolic population has apparently been derived from the ER, and appears to have been dislocated by ERAD in a non-ubiqutylated manner and then refolded, rather than being completely degraded, in the cytosol [49]. Thus certain proteins can uncouple from the final degradative step of ERAD. Protein toxins that retrotranslocate from the ER are believed to represent another family of proteins that fall into this category [35].

### 3.1. Ricin Entry into Mammalian Cells

Ricin, in common with other A-B family plant and bacterial toxins, enters mammalian cells by endocytosis after binding to cell surface components that inadvertently act as toxin receptors. In the case of ricin, which is a galactose-specific lectin, potential surface targets are all components with exposed β-1,4-linked galactose residues. Such components are typically abundant on mammalian cells (HeLa cells, for example, contain 3 × 10^7^ potential ricin binding sites [50]).

Some of the surface-bound ricin then enters the cell by endocytosis, the endocytic route utilised (e.g., clathrin-dependent or clathrin-independent endocytosis) being that followed by the surface component to which ricin opportunistically binds. Regardless of the endocytic route taken, the ricin that enters the cell is firstly present in early endosomes (EEs). From EEs, several possible fates are available. A portion of the ricin in EEs progresses via late endosomes to the lysosome, where it is proteolytically degraded. Some of the ricin in EEs enters recycling endosomes and is returned to the cell surface in a futile entry-exit cycle. A small portion of the ricin in EEs progresses to the *trans*-Golgi network (TGN), and this is the portion that is critical for cytotoxicity [51,52].

The retrograde trafficking pathway [53], which transports cargo from the TGN to the ER, usually via the Golgi complex, is the route taken by ricin (and other toxins that enter the target cell cytosol from the ER) to reach the ER lumen. In the case of ricin, molecular details of this trafficking step are largely unknown at present. The retrograde transport of Shiga toxin (STx) (another A-B toxin believed to enter the cytosol from the ER) has received more attention and is currently better understood [53,54]. It was originally assumed that ricin would use the same or similar components to those used by STx to achieve retrograde trafficking to the ER. However, recent studies are revealing differences, not in the ultimate destination of the retrograde pathway (namely, the ER), but in the molecular details of how different toxins are transported. A good example of this is shown by the comparison between the bacterial toxins STx and cholera toxin (CTx), both of which are eventually delivered to the ER. The small molecule Exo2, an inhibitor of a subset of Arf-GEF functions [55] strongly blocks egress of STx from EEs [56], but has little or no effect on CTx trafficking [57]. Furthermore, the clathrin associated Hsc70 co-chaperone RME-8 regulates endosomal trafficking of STx [58], but has no effect on the transport of CTx [59].

Regardless of the molecular details of the trafficking of individual toxins, each of the afore mentioned toxins, including ricin, reaches the ER as the final membrane-delimited compartment from which entry into the cytosol is achieved.

### 3.2. Pre-Dislocation Events in the ER

Once the ricin holotoxin reaches the ER lumen, RTA must be released so that it can retrotranslocate in a potentially catalytically-active conformation. The reductive separation of RTA and RTB is catalysed by protein disulfide isomerase [60,61] even in the oxidizing environment of the ER lumen [62].

RTA is believed to achieve ER export by subverting ERAD. Polyubiquitylation is a key feature for selecting proteins for proteasomal degradation, and the polyubiquitin chains are normally added to lysine redsidues. The catalytic A chains of toxins that enter the cytosol from the ER have an uncharacteristically low lysine content, and Hazes and Read [63] were the first to suggest that this low lysine content might allow the proteins to subvert ERAD for ER-to-cytosol transport. Experimental support for this contention in the case of RTA has been presented [64]. Introducing extra lysine residues into RTA increased its propensity for proteasomal degradation by rendering the modified RTA a better substrate for ubiquitylation [64]. Supplementing the A chain of CTx with lysines likewise increased the extent of its proteasomal degradation [65].

How is a native protein like RTA perceived by ERQC to be a substrate for the ERAD process? The answer appears to be that RTA partially unfolds in the ER. RTA contains a hydrophobic stretch of amino acids close to its *C*-terminus [9]. In the ricin holotoxin, this hydrophobic region is covered by RTB. When RTA is released from the holotoxin, this region becomes exposed allowing RTA to interact with the ER membrane. The first indication that such an interaction might promote RTA unfolding came from the demonstration that RTA did indeed unfold in the presence of liposomes containing a negatively-charged phospholipid [66]. Subsequently RTA was shown to unfold after interaction with ER-derived microsomal membranes, with negatively-charged phosphatidylserine the key membrane component in this interaction [67]. Furthermore, RTA inserts into the microsomal membrane at 37 °C and, predictably, it is the *C*-terminal hydrophobic region that enters the membrane [67]. Purified RTA is also thermally unstable, forming a molten globule at 45 °C [68], but even at 37 °C it is relatively unstable and prone to aggregation [69].

The driving force for RTA unfolding in the ER lumen appears to be thermal instability of RTA released from the ricin holotoxin, coupled with an ordered insertion into the ER membrane that results in partial unfolding of the RTA structure. It is speculated that this membrane-embedded form may mimic a misfolded protein that can then be dislocated from the ER in an ERAD-like manner. Prior to dislocation, ERAD substrates must be maintained in a soluble form, which may provide rationales for the requirement of co-chaperones of Bip [69,70], and the finding that the ERAD-specific mannosidase EDEM modulates ricin toxicity [71].

The A chain of STx also possesses a hydrophobic segment required for its dislocation when expressed in yeast ER [72]. A synthetic peptide containing this hydrophobic sequence interacts with lipid membranes [73,74]. While it is tempting to speculate that the interaction of STx A chain with membranes induces its partial unfolding, as is the case for RTA, this has not yet been experimentally demonstrated. 

### 3.3. Dislocation from the ER

RTA dislocation has been examined in *Saccharomyces*, plant systems and in mammalian cells, revealing a number of common aspects, but also showing that there are system-specific requirements.

RTA expressed exogenously in yeast and directed to the ER, dislocates and regains an active conformation in the cytosol, inhibiting yeast protein synthesis and causing a severe growth defect [70]. We have taken advantage of this, using a yeast library in which each non-essential gene has been individually knocked out, to identify gene knock-outs that alleviated this growth defect.

This work demonstrated that RTA does indeed subvert ERAD for export from the yeast ER, utilizing the integral membrane HRD E3 ubiquitin ligase complex for dislocation. Although RTA dislocation is strongly dependent on the core protein Hrd1p and its regulator Hrd3p, requirements for membrane-anchoring subunits of this complex and the accessory factors that optimize its performance are intermediate (e.g., Der1p, the yeast homolog of derlin-1) or minor and it is independent of the E3 activity of Hrd1p [70], indicating that canonical ubiquitylation does not occur during dislocation. Consistent with this, RTA is not extracted from the yeast ER by Cdc48p or its ubiquitin-handling co-factors [70]. Rather, the RTA extraction motor appears to be the Rpt4p subunit of the proteasomal cap, which had previously been shown to play a role in the extraction of other substrates in conjunction with Cdc48p [46]. Curiously, dislocated RTA is not degraded by the proteasomal core, at least in yeast, even though there is significant degradation of the toxin during pulse-chase experiments [70].

Thus RTA appears to be dislocated in yeast promiscuously through non-anchored, non-optimised dislocons, and it avoids proteasomal degradation. In marked contrast, a mutant form of RTA that is unable to fold into the native conformation acts as a *bona-fide* ubiquitylated and *N*-glycosylated ERAD substrate that is extracted in a Cdc48-dependent manner, de-glycosylated by peptide *N*-glycanase [76,77], transferred to the proteasome by Rad23p and degraded by the proteasomal core [70].

ER dislocation of RTA in plant cells is likewise independent of ubiquitylation, although there appears to be a role for Cdc48 [75], and a proportion is degraded by proteasomes [78].

The RTA dislocation process in mammalian cells has received little attention to date. The translocon component Sec61p may be involved [79,80]. A role for the mammalian HRD complex regulator SEL1L (the mammalian equivalent of Hrd3p) has been described for the dislocation of RTA [81], suggesting that RTA utilises components of the ERAD machinery to enter the mammalian cytosol. However, derlin-1, which is a component of the dislocon in mammalian cells [82,83], does not appear to have a role in the dislocation of either RTA [71,81] or the CTx A chain [84] and there appears to be a role for the mammalian equivalent of Cdc48, p97 [85]. Furthermore, proteasomal inhibition sensitises cells to ricin [64,80] and retards loss of attenuated versions of RTA after direct expression in the mammalian ER [81].

### 3.4. Post-Dislocation Events in the Cytosol

Because RTA co-opts ERAD components by partially unfolding in the ER lumen, it enters the cytosol as a misfolded protein and must therefore refold to recover catalytic activity to become active against ribosomes. *In vitro*, RTA unfolded to a molten globule structure recovers catalytic activity in the presence of substrate ribosomes [68]. In mammalian cells however post-dislocation scrutiny by the cytosolic chaperone Hsc70 is required for RTA refolding [69]. *In vitro*, Hsc70 can prevent aggregation of heat-denatured RTA, so one *in vivo* role for this chaperone could be to aid the solubility of dislocated RTA thus allowing it to undergo ribosome-mediated refolding. Alternatively, the role of Hsc70 may be to stabilise RTA in the cytosol by masking the hydrophobic region that interacts with membranes. 

**Figure 2:**
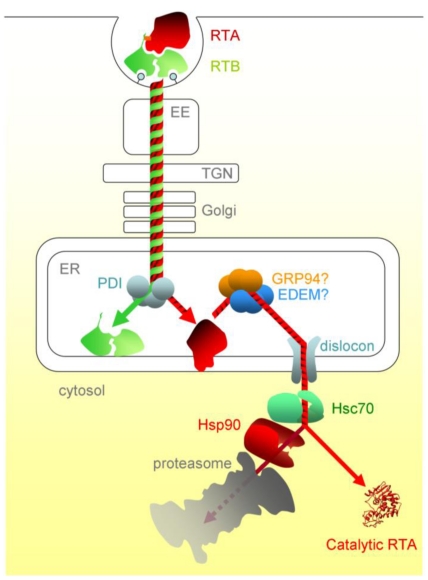
The cytotoxic route of ricin in mammalian cells. After entering the cell by endocytosis, ricin traffics via vesicular carriers through the early endosomes (EE) and the, *trans*-Golgi network (TGN) to the ER. ER processing events include separation of RTA and RTB, interaction of RTA with the ER membrane and likely interactions with luminal chaperones prior to dislocation. Post-dislocation triage by cytosolic chaperones is thought to enable a proportion of the dislocated RTA to refold. PDI, protein disulfide isomerise; EDEM, ER degradation enhancing alpha-mannosidase I-like protein; GRP94, 94 kDa glucose regulated protein.

The concentration of Hsc70 co-chaperones in the cytosol modulates the cell’s ability to aid RTA refolding, with some (HIP and BAG-2) promoting toxin refolding, while others (such as the proteasome-engaging BAG-1) promote degradation. In addition, there is a sequential chaperone triage in the cytosol, where a proportion of RTA is passed, via the Hsc70-Hsp90 organising protein HOP, to the Hsp90 chaperone. From here, the net fate of RTA is inactivation, presumably by proteasomal degradation. Although RTA is not ubiquitylated during dislocation, a low level of cytosolic ubiquitylation can occur, albeit the E3 responsible is not yet known [70]. *In vitro*, RTA can be inefficiently ubiquitylated in the presence of Hsc70 and the cytosolic CHIP E3 ubiquitin ligase, and this can be improved by mimicking the sequential triage by adding HOP and Hsp90, suggesting that the Hsp90 interactions inactivate RTA by stimulating cytosolic ubiquitylation [69]. Thus a network of chaperones can determine the fate of RTA in the cytosol by regulating the competing processes of refolding and ubiquitin tagging. This may provide a rationale for why the inhibition of proteasomal degradation slightly sensitises cells to intoxication by ricin, whose toxic subunit is not ubiquitylated during its dislocation process [64,80]. Ricin entry and trafficking in mammalian cells is illustrated schematically in Figure 2.

## 4. Conclusions

During its synthesis in the producing plant cells ricin traffics in an anterograde manner from the ER, via the Golgi complex, to the vacuole. When it intoxicates mammalian cells this protein undergoes retrograde transport from the cell surface to the ER, from where it is translocated into the cytosol. Ricin thus crosses the ER membrane twice, from the cytosol to the ER lumen during its synthesis and from the lumen to the cytosol as it enters target eukaryotic cells: an interesting and unusual occurrence for this interesting and unusual protein.
